# The usefulness of on-site physical therapy-led triage services for professional orchestral musicians – a national cohort study

**DOI:** 10.1186/1471-2474-14-98

**Published:** 2013-03-19

**Authors:** Cliffton Chan, Tim Driscoll, Bronwen Ackermann

**Affiliations:** 1 Discipline of Biomedical Science, Sydney Medical School, P.O. Box 170, Lidcombe, NSW, 1825, Australia; 2 School of Public Health, Sydney Medical School, Camperdown, 2006, Australia

**Keywords:** Injury management, Performance-related musculoskeletal disorders, Performing arts medicine, Physical therapy, Prevention

## Abstract

**Background:**

Australian professional orchestral musicians reported a lifetime prevalence of musculoskeletal injuries that had interfered with playing at 84%. Physical therapy-led triage clinics may be a practical method to manage the impact of high performance-related musculoskeletal disorders (PRMDs) in professional orchestral musicians. This study aimed to: a) collect information on presenting injuries, b) determine the participant’s provisional diagnosis, c) evaluate uptake of an on-site triage service, d) measure participant satisfaction, and e) identify factors influencing attendance.

**Methods:**

Eight triage sessions were run on a fortnightly basis during a designated lunch break between rehearsal calls in seven premier symphony orchestras in Australia; a total population of 483 musicians. The participants received one or a combination of: a) education and advice relating to their provisional diagnosis, b) basic acute management and/or c) a referral to a suitable medical practitioner or allied health professional for further consultation or treatment. A three-month follow-up questionnaire was completed and a qualitative narrative themes-based analysis was undertaken to summarise participant and physical therapist feedback. Uptake, participant satisfaction and factors influencing attendance were measured.

**Results:**

99 initial consultations (83 individuals) were conducted with more females (61%) utilizing the service than males (49%). The most common injury complaints were in the shoulder (22%), neck (18%), upper back (18%), and hand (8%). 66% of these were diagnosed as PRMDs. Of these injuries, 94% were considered preventable, 93% continued to affect playing, 68% were severe requiring a referral for further management, and 46% were recurrent. The advice at the triage service was rated as helpful or very helpful by 79% of the musicians, whilst 68% responded they were likely or very likely to continue to use the service if it was offered in the future. Of the participants that followed through with the referral advice, 67% reported that the referral advice was helpful or very helpful. Musicians’ and physical therapists’ written feedback indicated their acknowledgement for the need of this service. The main suggestions for improving attendance were increasing the music-specific physical therapy knowledge of therapists and overcoming competing time demands.

**Conclusion:**

On-site health services for musicians may facilitate better injury management by providing immediate and specific health advice.

**Trial registration:**

ACTRN12612000220864

## Background

Professional musicians have been termed “a working population in crisis” due to the high rates of occupational injuries that have been widely reported over the last 30 years [1-3]. There is a strong body of evidence recognising the prevalence, incidence and types of performance-related musculoskeletal disorders (PRMDs) in this population [4-6].

Australia has a national strategy to reduce musculoskeletal workplace injury by implementing injury prevention and control interventions [7]. The high injury rates in Australian professional orchestras have resulted in increased workers’ compensation claims and insurance premiums as well as associated costs from sick leave, creating a substantial burden on orchestral organisations [8]. It has long been recognised that the long hours of daily training and complex neuromuscular skills involved in playing an instrument at a professional level are comparable to training and performance demands of elite athletes [9,10]. A striking contrast between the two groups is the far greater prevalence of low-load overuse injuries in musicians, unlike the high-velocity or high-impact injuries in athletes, however both groups are prone to overuse injuries with specific postural and muscle imbalances related to their task demands [11-14]. These highly sophisticated training and performance challenges may lead to dysfunctions or secondary injury pathologies emerging as a result, including inefficient compensatory movement patterns, joint hyper or hypo-mobility and nerve entrapments [6,15,16].

A recent study of Australian professional orchestral musicians revealed that 84% reported a lifetime prevalence of musculoskeletal injuries that had interfered with playing [17]. An average of 50% of these players had pain or injury for at least one week at the time of the survey, with the majority of these experiencing symptoms for longer than twelve weeks. In contrast, only 5% of the Australian population in other occupations report work-related injuries over a one-year period [18]. The aforementioned injury prevalence rates may be even higher than the reported figures, as professional musicians typically under-report performance-related problems [19,20].

Triage clinic services aim to provide patients with an appropriate and timely clinical pathway for specific health issues [21,22]. They have proven successful in many medical fields [23,24], and more recently in managing injuries within the elite athletic and dance populations [25,26]. In Australia, professional orchestral musicians do not have the same medical support network as the sports and dance populations for their performance-related problems [27], with none of the professional orchestras currently engaging regular services of a health professional. As a consequence of little or no health education or support during their training years as music students, many musicians have a lack of understanding of injury causation and recognition and optimal health practices. This can often lead to feelings of professional inadequacy or shame associated with developing or sustaining an injury [19,20,28].

There is no formalised musicians’ health training program for health professionals in Australia, and this may lead to non-specific injury management advice without a clear rehabilitation direction to regain full music performance capacity. One way of addressing these barriers is to provide easily accessible healthcare advisory services run by senior clinicians with an interest in working with musicians. Based on a previous report of the efficacy of a brief intensive physical therapy-led triage service for professional orchestral musicians, the availability of a regular triage service may allow earlier identification and management of performance-related musculoskeletal disorders (PRMDs) [29].

The aim of this study was to trial a nationwide triage clinical service in professional symphony orchestras to: a) record the duration, location and perceived cause of presenting injuries b) determine a provisional diagnosis based on the injury assessment, c) evaluate uptake of the service, d) measure participant satisfaction with the service, and d) identify factors influencing attendance at the service.

## Methods

### Pilot study

A pilot trial was first conducted to evaluate feasibility and potential usefulness of the triage service prior to expanding this research study to a larger national population of professional musicians. The trial was conducted at one of the premier symphony orchestras in Australia and which had 96 professional orchestral musicians. Over the trial period, 15 participants (four male and eleven female) with a mean age of 44.7 years (S.D. 11.0) attended the triage service. These musicians were: five violinists, four violists, two double bass players, one cellist, one flautist, one oboist and one percussionist. Four triage sessions were conducted three weeks apart, with each session lasting approximately one and a half hours. Consultation duration ranged from 10–25 minutes.

Feedback from these musicians indicated that the service was useful and a longer duration of the trial would have been desirable. The physical therapists also suggested increasing the overall number of triage sessions (from four to eight) as well as their frequency (from every three weeks to fortnightly). The duration of each consultation was highly variable in the pilot trial - this was standardised to 15 minutes for the main study. The assessment record was slightly modified to allow more detailed information to be collected; e.g. adding a more detailed head and hand-specific section, and increasing the use of check boxes where possible to expedite the documentation process.

### Study participants

#### Musicians

Orchestral musicians were recruited through email notification sent out by the orchestra representatives and flyers displayed on orchestra noticeboards. All musicians employed in one of the seven remaining professional symphony orchestras could participate in this study; this was a total of 483 musicians. Musicians with an injury that was the subject of a workers’ compensation claim were excluded and their data was not collected in this study. This was because workers compensation clients in Australia follow an injury management plan prescribed by a specially appointed healthcare team [30,31].

#### Physical therapists

Expression of interest flyers were sent to 19 senior clinicians around Australia who had previous experience with musician studies or had a special interest in musician’s health. Fourteen physical therapists were recruited as a result; each had at least seven years of clinical experience and a post-graduate qualification in occupational or sports physical therapy.

### Study protocol

Orchestra management allocated a list of triage dates and venue/room facilities based on their rehearsal schedules and the study’s research requirements. The authors matched these dates and venue locations with the physical therapists’ availability. Eight triage sessions, a fortnight apart, were then organised with each orchestra. Participants could sign-up to the service (anonymously if they wished) or attend on the day if there were available appointments. The triage service was open for one hour during a designated lunch break between rehearsal calls. This was to maximise exposure of the service because attendance appeared to be higher during the lunch hour in the pilot trial. Physical therapists were advised to keep appointments approximately 15 minutes in duration. The triage services were conducted between January and December 2011 and all follow-up surveys were completed by April 2012.

**Figure 1:**
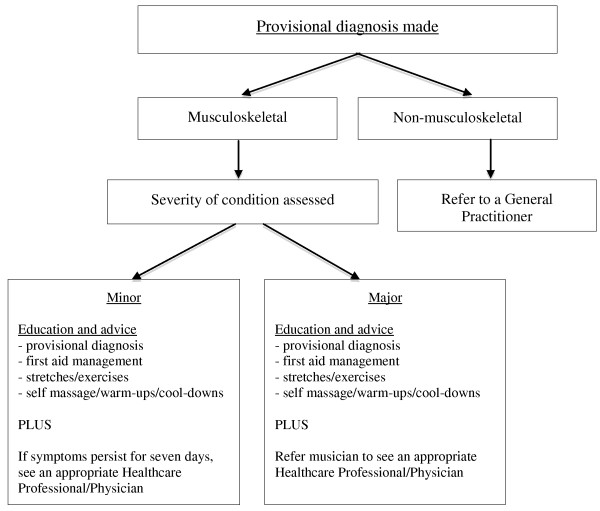
Management protocol of the triage clinic.

Physical therapists were trained to conduct a standardised protocol including the use of the assessment form (Additional file 1), undertaking an injury assessment, recording characteristics of the injury, making a provisional diagnosis, and providing education and advice. After the completion of the physical assessment, physical therapists were asked to use their clinical experience to determine a provisional diagnosis. Physical therapists were also asked to define the injury as *major* or *minor;* where *major* was used when the presenting injury required more in-depth management by a healthcare professional and *minor* when an injury was not likely to need further management. The severity of the injury determined whether the participants received one or a combination of: a) education and advice relating to their provisional diagnosis, b) basic acute management and/or c) a referral to a suitable medical practitioner or allied health professional for further consultation or treatment (Figure 1).

To assess the long-term effects of this method of triage consultation, a follow-up survey was emailed three months afterwards to evaluate whether musicians had followed-up on the advice given. If no initial response was received, another email was sent and then two telephone calls were made one week apart before the participant was considered a ‘non-responder’.

### Outcome measures and data analysis

The participant’s demographic information, history and characteristics of injury (i.e. chronicity, preventability, performance-relatedness) and injury management information were recorded at the time of consultation. A standard musculoskeletal classification of injury chronicity was used (i.e. acute, chronic recurring, chronic) [32]. An injury was determined by the consulting physical therapist to be preventable if the therapist perceived that the injury could have been avoided through strategies such as strength and conditioning training [33]. A musculoskeletal disorder was considered performance-related if the injury occurred during or immediately after playing and the musician specified that instrumental playing was the main contributor to their injury [34]. In the follow-up survey, three questions were asked in three main areas. These concerned: (i) the participant’s opinion on helpfulness of the service (ii) their likelihood of using the service again and (iii) the usefulness of the referral advice. A five-point scale was used, as well as open-ended questions. In addition, space was left for musicians to make any other comments to the research team that they felt were relevant to the project. The physical therapists who provided the service were contacted by either phone or email at the end of the trial and asked for feedback on their experiences.

Quantitative analysis was conducted using statistical software SPSS 19.0. Descriptive statistics were obtained for the participant characteristics, demographics and satisfaction rates, and an independent t-test was performed on age by gender. One of the investigators (CC) read the follow-up feedback received and undertook a method of qualitative narrative analysis, as described by Braun and Clarke (2006), to identify major themes that emerged [35].

### Ethics

The University of Sydney Human Research Ethics Committee approved this study (HREC 12523). All participants gave written informed consent before data collection began.

## Results

### Participants

Eighty-three professional orchestral musicians, 35 males and 48 females, attended the triage services available at their orchestras. Seventeen per cent (83/483) of eligible musicians had at least one consultation during the trial. There were 109 consultations undertaken in total, of which 10 were return consultations. The age range of the male musicians (45.4 yrs ± 10.8) and female musicians (42.7 yrs ± 10.3) were similar (t = 1.19, p = 0.236). Although more females attended the clinic overall, the percentage of female to male upper string players (48% females vs 46% males) and brass players (9% females vs 7% males) were similar (Table 1). The other instrumental groups showed a slight gender difference: lower string players (16% females vs 22% males) and woodwind players (24% females vs 17% males).

**Table 1:** Participants who attended triage services - by instrument played, body region of injury and gender

**Body regions**	**Upper strings**		**Lower strings**		**Woodwind**		**Brass**		Percussion* / harp^ / Keyboard~		**Total by body region**	
	**Male**	**Female**	**Male**	**Female**	**Male**	**Female**	**Male**	**Female**	**Male**	**Female**	**Male**	**Female**
	**n = 19**	**n = 28**	**n = 9**	**n = 9**	**n = 7**	**n = 14**	**n = 3**	**n = 5**	**n = 3**	**n = 2**	**n = 41**	**n = 58**
Head & face	1	1	-	-	-	-	-	-	-	-	1	1
Neck	2	8	1	-	-	6	-	1	-	-	3	15
Upper back	4	6	1	2	-	3	1	1	-	-	6	12
Lower back	-	1	1	-	-	1	1	-	-	-	2	2
Shoulder	6	7	3	-	3	-	1	-	1*	1^	14	8
Elbow	-	1	1	-	1	-	-	1	1*	-	3	2
Forearm	1	1	1	1	-	-	-	-	-	-	2	2
Wrist	3	-	-	-	-	-	-	-	-	1~	3	1
Hand	-	1	1	3	-	2	-	1	-	-	1	7
Thumb	-	1	-	2	1	-	-	-	-	-	1	3
Hip	-	-	-	1	1	1	-	1	-	-	1	3
Knee	2	1	-	-	1	1	-	-	1*	-	4	2

### Injury characteristics

The initial injury consultations are summarised in Table 1, arranged by instrumental groups, body regions and gender. Specific information on the characteristics of the injuries, such as chronicity, preventability, continual effect on playing, prevalence and severity, is presented in Table 2. Sixty-six per cent of injuries were considered to be PRMDs. Provisional diagnoses of these injuries revealed six main categories: 1) muscular strain, tendinopathy or enthesopathy due to overuse (37%); 2) poor postural control (29%); 3) joint hypomobility (22%); 4) nerve entrapment (7%); 5) lack of full recovery post-operatively or after a previous injury (3%); and 6) joint hypermobility (2%). Pain in the neck and shoulder had the largest difference between gender, with more female instrumentalists presenting with neck problems (26% female vs 7% male), and more males reporting shoulder problems (14% female vs 34% male).

**Table 2:** Prevalence rates and characteristics of musculoskeletal injuries reviewed at the triage services

**Characteristic of injury**		**All Injuries *(n = 99)***	**PRMDs *(n = 65)***
Chronicity	*Acute (0–2 wks)*	30%	26%
	*Chronic Recurring*	48%	46%
	*Chronic (>3 mths)*	22%	28%
Currently Affects Playing		67%	93%
Preventability		83%	94%
Severity	*Major*	61%	68%
	*Minor*	39%	32%

### Follow up survey data

Seventy-two participants (87%) responded to the three-month follow-up questionnaires. Of the 11 participants lost to follow-up, six were overseas, one was on long service leave and the reason for no response of the remaining four was unknown. Based on those who responded, 79% of participants rated the advice at the triage service as helpful or very helpful. Sixty-eight per cent of participants responded they would be likely or very likely to continue to use the service. Of the 39 respondents who were provided with a referral, 61% had followed through with this advice. Sixty-seven per cent of these participants reported that the referral advice and information provided were useful or very useful (Table 3). The reasons for the 15 participants who did not follow through with the referral advice were: already planned to see their usual healthcare professional (7), injury got better post-triage consultation (5) and only used the triage service to get a second opinion (3).

**Table 3:** Summary of results from the three-month follow-up response

**Rating**	**Helpfulness of advice**	**Likelihood of future use**	**Usefulness of referral**	
		***n = 72***	***n = 72***	***Participants provided with referral advice n = 39***	***Participants who followed through with referral advice n = 24***
1 Not at all	3%	1%	18%	8%
2	5%	3%	5%	8%
3 Not sure	10%	25%	33%	13%
4	26%	30%	15%	25%
5 Very	53%	38%	26%	42%
No response	3%	3%	3%	4%
**Total**	**100%**	**100%**	**100%**	**100%**

### Qualitative feedback

Ninety-one per cent of the participants and all fourteen physical therapists provided written feedback about the triage service. Major themes identified through the qualitative methodology of narrative analysis are outlined below. Note that the percentages do not total to 100% because some participants and physical therapists gave feedback pertaining to more than one theme.

a) Acknowledgement

Overwhelmingly, the biggest response from participants and physical therapists was to express appreciation of and the need for the availability of the triage service.

i. Seventy-seven percent of the participants were grateful to have on-site and regular access to physical therapy assessment and advice.

“…the availability of different resources is a welcome scenario as a necessary adjunct to our everyday “given” work conditions.”

“Often pain is accepted as part of the job. If triage was readily available I would probably be more likely to enquire about certain pains…”

ii. All physical therapists appreciated their opportunity to work with professional orchestral musicians; a population that has specific injuries that they do not regularly see in clinic.

“Overall I found the involvement in … triage clinic to be professionally stimulating and rewarding. As you would know, the musicians are a great population to work with.”

b) Longer consultation and treatment time

i. Eighteen per cent of the participants stated that the short triage consultations were useful but they would have liked to expand the service to include manual treatment.

“I do think it is great to have this available for consultation, however I think that a more hands on approach is often required.”

ii. Three physical therapists reported that if the consultation was longer, a more comprehensive service could have been provided.

“Tight time frame…20-30 minute session length would have been ideal.”

c) Perceived lack of specific music physical therapy experience

i. A small group of participants (6%) stated that the physical therapists involved in the trial required more specific music, instrumental and musician-specific injury knowledge.

“Good to have someone there to see about the injury - might be good to have someone who is more familiar with instrument-specific problems.”

ii. Two physical therapists reported that they would have benefitted from more post-graduate musician-specific physical therapy training. Nonetheless, they greatly valued the experience they received from the trial.

“This trial was great to be involved in since I do not see musicians very often… It would be great if we could get access to some musician physiotherapy training…”

d) Fear of injury exposure.

i. Some participants (4%) expressed their discomfort with seeing an on-site physical therapist due to fear of other parties discovering their injury.

“I think that a stigma exists where musicians don’t want to talk about their injuries, nor see a physio about it on worksite.”

## Discussion

The onsite physical therapy triage clinics were well received by attending musicians, with most players indicating that they felt the service to be worthwhile. Nearly one fifth of all orchestral players eligible to attend utilised the service, and the feedback indicated that such clinics, held regularly and offering more services and longer sessions, would be of even greater benefit to the players. The musculoskeletal injuries experienced by the professional orchestral musicians were mostly of a performance-related nature, which required further referral to an appropriate healthcare professional for management.

### Demographics and characteristics of musicians and injuries

The age distribution of musicians presenting for consultation was representative of the Australian orchestra population [17]. A larger proportion of female musicians utilized the service, perhaps reflecting the higher reported prevalence of musculoskeletal disorders in female musicians [4,5] or the increased likelihood of females using healthcare services in general, particularly for preventative health and diagnosis [36,37]. Consistent with previous injury prevalence research [4,5,17,38], the main reported problems in musicians who attended the triage clinics were located in the shoulder, followed by the neck, upper spinal region and hand. Upper string players had the highest number of consultations. Gender discrepancies in harpists, keyboard players and percussionists attending the service reflected differences in the professional orchestral musician population rather than a gender difference in seeking health advice. A higher proportion of male lower string players and female woodwind players were the main gender-specific instrumental group difference seen in musicians attending the triage service. However, this difference should be interpreted with caution due to the small sample numbers. Gender appeared to have had an impact on where pain was reported, with more complaints reported in the neck by females as in previous research but problems in the shoulder were relatively more frequent in males in this study [4,5]. This study only recorded information from a sixteen week time period, and this may have influenced these findings in comparison with 12-month or lifetime prevalence studies that are of a longer time frame [5,6].

The majority of the injuries seen were diagnosed by the physical therapists as musculotendinous strains/tendinopathies or joint hypomobility and were mostly attributed to poor postural control. These problems can be prevented and are generally managed well with appropriate education and advice and physical therapy clinical interventions such as strength and conditioning exercises and manual therapy [39-41]. Of significant clinical relevance is the fact that nearly half of all performance-related injuries seen at triage were chronic recurrent injuries. A recent study on this cohort of Australian musicians revealed that of those musicians who had a past musculoskeletal injury, less than 50% reported they had fully recovered from the injury [17]. It is believed that healing tissues exposed to repeated mechanical stress (e.g. instrumental playing) impedes full recovery and increases the likelihood of a subsequent injury [16,42]. This highlights the importance of providing appropriate healthcare services for musicians to encourage earlier diagnosis and treatment of performance-related musculoskeletal injuries.

### Intervention uptake

Ackermann and colleagues [17] found that approximately half of the Australian professional orchestral musician population were experiencing a musculoskeletal injury at any one time. If this is the case, approximately one third of this injured cohort experiencing a musculoskeletal disorder used the triage service. Considering that some musicians already have preferred healthcare providers, the uptake of the triage service was very encouraging in terms of increasing accessibility of such advice for a large group of musicians. However, the uptake rate could be further improved by removing some barriers to triage accessibility. The investigators had to work closely with each orchestra’s management to schedule the triage sessions around the musicians’ irregular and repeatedly changing rehearsal roster. This then needed to match the local physical therapist’s availability. Also, musicians often have committee meetings and administrative duties during the lunch hour that preclude them from attending scheduled triage sessions. For example, one solution to these barriers may be by scheduling the triage sessions during rehearsals and arranging permission for injured musicians to be able to attend the triage service during rehearsal hours [43].

### Intervention satisfaction and feedback

Musicians were greatly appreciative of the convenience of the triage service being available at work and that orchestral management and the investigators made an attempt to plan the service around their rehearsal schedule and meetings. Further, they felt that having a cost-free injury advisory service was an attractive and welcomed resource for professional orchestral musicians; this allowed them to seek earlier advice on injuries than would have been the case without the service. This triage service for professional orchestral musicians providing education and advice for their injuries was perceived to be useful and, based on their responses, would be used again if offered in the future. Most of the musicians who were provided with referral advice due to the need for further clinical intervention found this information to be valuable. The physical therapists found the experience of working with the musician population rewarding and that it positively challenged their knowledge and clinical reasoning skills. They expressed an interest in further training in this area of physical therapy to improve their ability to manage some of the specific musician problems that had presented to the triage clinic.

The main goal of this triage service was to assess the presenting problems of professional orchestral musicians and provide an early and immediate management pathway. Feedback from the musicians and physical therapists expressed the need for this service, but also suggested that longer consultation times would be of even greater benefit by providing a full physical therapy consultation. This provision of full health care services at the orchestra premises could be trialled in future research projects.

### Other factors that influenced attendance

Since there is sparse qualitative literature on health management of professional orchestral musicians [44], the participants’ feedback on the triage service was important in understanding in more depth the perceived usefulness of the triage services. While as an overall group players were satisfied with the service, a small percentage of musicians commented that the limited music and instrumental knowledge of the attending physical therapist hampered their ability to identify the cause of performance-related problems. This meant that they were not provided with specific enough advice and education to address the more complex playing-related issues. This situation may affect the musician’s ability to establish trust with the healthcare provider and in turn have a negative effect on advice and referral adherence [45,46]. Despite the recruitment of experienced physical therapists with post-graduate specialist degrees in occupational or sports physical therapy, these skills were inadequate when the injuries required instrument specific knowledge to manage optimally. There is a need for specialised training in musician-specific conditions and music performance requirements for physical therapists to enhance their provision of healthcare to musicians [41,47].

Exposure of performance-related injury within musical communities has been previously documented to be associated with negative connotations related to the performers’ abilities as well as fears of employment termination [20,48]. Therefore, as reported by one musician involved in the trial, there may have been some reluctance from some players to report an injury or seek injury advice if they felt this would be known to orchestral peers and management. The persistence of these negative injury stigmas need to be challenged by promoting health education and raising injury awareness in both orchestral management and musicians.

### Strengths and limitations

This is the first study to specifically evaluate an intervention aimed to facilitate easy access to quality healthcare for professional orchestral musicians. The service was strategically conducted at the orchestra premises, with the private consultation room located as far away as possible from common areas. The intervention was delivered over eight fortnights to allow musicians to familiarise themselves with the service and attending physical therapists to increase the likelihood of attendance over a sufficient duration to capture the typical characteristics of performance-related injuries. A limitation of this study was that extrapolation of the results from this study to the whole of Australia or to other musician populations should be made with caution since the sample size was relatively small and the study conducted over a limited time frame. Due to the nature of triage clinics, selection bias (i.e. self-selection participation) may also have influenced results. Future studies could look into screening clinics for musicians, in particular targeting new employees such that longitudinal prevalence studies and prevention trials could be conducted.

## Conclusion

A trial of an on-site physical therapy-led triage service in a national cohort of professional orchestral musicians indicated that this is a useful and well-accepted resource amongst professional orchestral musicians. Typical injured body regions included the shoulder, neck and upper spinal regions, with most of the injuries being chronic and recurrent and preventable. Future studies could include investigating the effectiveness of a longer duration of regular on-site physical therapy service in decreasing the intensity and frequency of PRMDs, as well as evaluation of the impact of incorporating more music-specific injury prevention and management training of available service providers.

## Competing interests

The authors declare that they have no competing interests.

## Authors’ contributions

CC participated in study design, coordination, data collection, data analysis and paper writing. TD participated in study design and paper writing. BA participated in study design, data analysis and paper writing. All authors read, revised the manuscript and approved the final version.

## Pre-publication history

The pre-publication history for this paper can be accessed here:

http://www.biomedcentral.com/1471-2474/14/98/prepub

## Supplementary Material

Additional file 1 Triage Assessment Form. Click here for file
