# Supplementary material for: The usefulness of on-site physical therapy-led triage services for professional orchestral musicians – a national cohort study
Source: BMC Musculoskelet Disord. 2013 Mar 19;14:98. doi: 10.1186/1471-2474-14-98 (PMC3614523; doi:10.1186/1471-2474-14-98)
Supplement: Additional file 1 — Triage Assessment Form. [file 1471-2474-14-98-S1.pdf]

Supporting sustainable careers in orchestral musicians through Occupational Health and Safety initiatives

Triage Assessment Form

**Date:**	**D.O.B:**
**Name:**	**Instrument:**
**Date of Injury:**	**Where injury occurred:**
**Area of injury:**<br>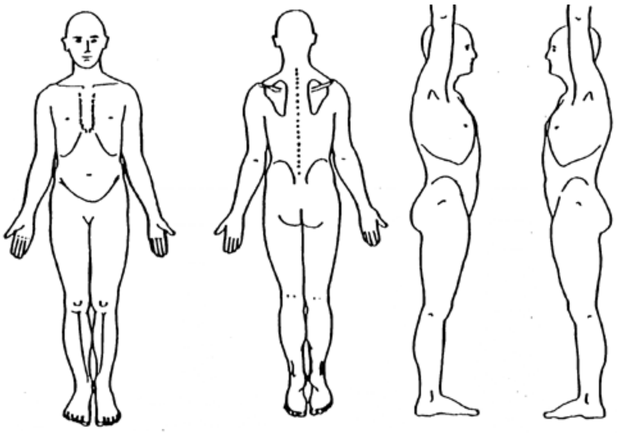<br>Acute / Chronic recurrent / Chronic	**Area of injury (face and hand detail):**<br>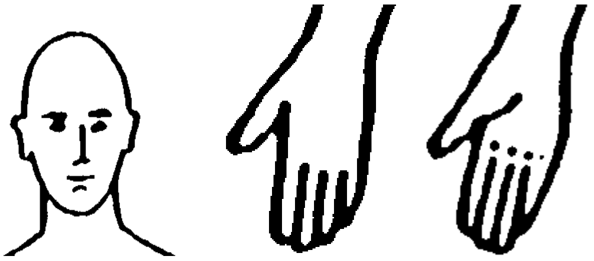<br>Acute / Chronic recurrent / Chronic
**Current History:**	**Relevant Past History:**
**Observation:**	**Impression of injury (provisional diagnosis):**<br><br>**Was injury preventable? Y / N ……………………**<br><br>**Performance-related? Y / N ………………………**
**Affecting playing?: Y / N**<br>Specify:	**Advice given:**
**Further referral?: Y / N**<br>Specify:	**Consent to Follow-Up? Y / N**<br><br>**Email/Phone: ……………………………………..**<br><br>**Examined by:**
